# Improvement in Hearing Loss Following Posterior Fossa Arachnoid Cyst Fenestration: A Case Study

**DOI:** 10.7759/cureus.51750

**Published:** 2024-01-06

**Authors:** Gavin M Lockard, Keaton Piper, Jay I Kumar, Nicole Riddle, Oliver Flouty

**Affiliations:** 1 Neurosurgery and Brain Repair, University of South Florida, Tampa, USA; 2 Pathology and Cell Biology, University of South Florida, Tampa, USA

**Keywords:** hearing loss, headache, excision, fenestration, infratentorial, arachnoid cyst, posterior fossa

## Abstract

Arachnoid cysts are abnormal intradural collections of cerebrospinal fluid. For posterior fossa arachnoid cysts (PFACs), symptoms vary greatly, often relating to cranial nerve impingement and/or hydrocephalus. Literature on long-term symptomatic and radiographic follow-up of PFACs is lacking. This case study describes a 32-year-old man who presented with headaches and left-sided hearing loss and was found to have a large left-sided cerebellopontine angle arachnoid cyst with syrinx and ventriculomegaly. After PFAC fenestration and excision, his headaches resolved and his hearing markedly improved. At the one-year postoperative evaluation, symptom improvement persisted, and MRI demonstrated a stable decreased cyst and near-complete resolution of his syrinx.

## Introduction

Arachnoid cysts are intradural collections of fluid often arising from congenital or traumatic etiologies [1]. When located in the posterior fossa, they may precipitate a heterogeneous constellation of symptoms including headache, nausea/emesis, hearing loss, tinnitus, or otherwise, often relating to the cranial nerves involved [1]. Patients may also develop structural abnormalities such as syringomyelia, hydrocephalus, and tonsillar herniation. Surgical options aim to communicate the cyst with spaces amenable to CSF absorption via fenestration or shunting [1]. Offering surgery remains a contentious decision, previously based on patient preference and anecdotal experience. Previous surgical indications have included progressive symptoms or mass effects [1], but the nuances of what size and which symptoms should influence surgical decisions or are likely to improve are still unclear. This case report aims to provide long-term clinical and radiographic outcomes of posterior fossa arachnoid cyst (PFAC) fenestration, in a case that produced complete resolution of headaches and marked improvement in hearing loss, as well as a decrease in cyst size and near-complete resolution of syringomyelia on MRI.

## Case presentation

Preoperative course

**Figure 1:**
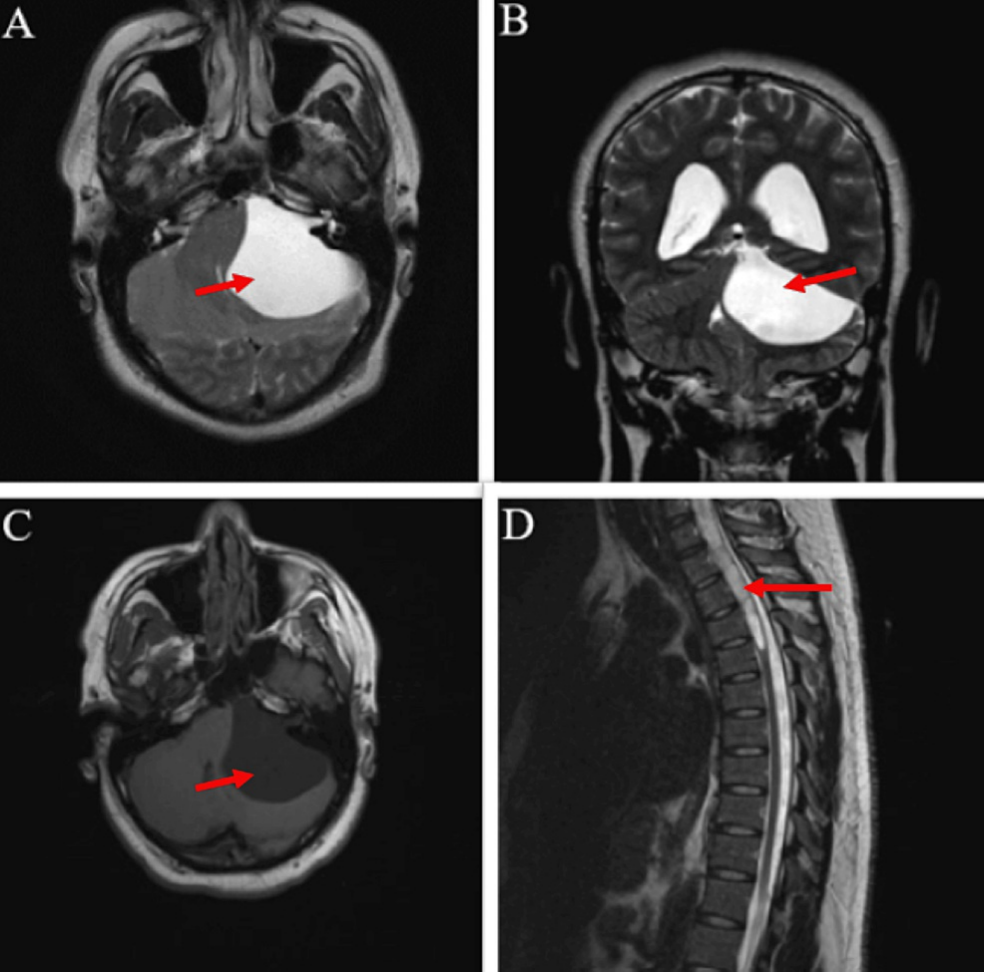
Preoperative MRI with arrows demonstrating the PFAC at the left CPA with mass effect on the midbrain, pons, and cerebellum (a) axial T2; (b) coronal T2; (c) axial T1; (d) sagittal T2 with arrow demonstrating syringomyelia

A 32-year-old man without pertinent past medical history presented with 16 months of progressive headaches worsened by the Valsalva maneuver and near-complete left-sided hearing loss. He also complained of left lower extremity weakness, resulting in decreased agility and poor gait. A physical exam demonstrated bilateral patellar hyperreflexia, no serviceable left-sided hearing on the finger rub test (audiogram not obtained due to timing and access), and an unstable gait. His neurological exam was otherwise intact, including cerebellar signs, motor strength, and other cranial nerves. Fundoscopy was not completed; however, the patient had no visual complaints. MRI demonstrated a large left infratentorial arachnoid cyst located in the cerebellopontine angle (CPA), measured at 6.3 x 4.6 x 4.5 cm, with a mass effect on the brainstem and cerebellum. There was also partial effacement of the fourth ventricle with mild lateral and third ventriculomegaly. He had a secondary Chiari malformation type 1 with a cervicothoracic syrinx extending from C2 to T5 and cerebellar tonsillar herniation 6 mm below the foramen magnum (Figure 1). There was no diffusion restriction, as confirmed by a neuroradiologist. Given his progressive headaches refractory to over-the-counter medication, gabapentin, and lifestyle modifications, he underwent a retrosigmoid craniotomy for cyst fenestration and a near-complete cyst wall excision.

Surgical procedure

The patient was positioned laterally for a retrosigmoid craniotomy, utilizing neuronavigation to optimize a “c”-shaped incision, ~ 3 cm posterior to the pinna. After opening the skin and performing a ~ 3 cm craniotomy posterior-inferior to the sigmoid and transverse sinuses, the dura was opened in a “c”-shaped manner with an incision bifurcating the dural flap toward the anterior-superior corner. After gentle retraction of the cerebellum posteriorly, the cyst was evident, with all cranial nerves displaced anterior-medially. Given the size, the cyst was immediately incised with a complete resection of the anterior and lateral walls, allowing good visualization of the cranial nerves. The prepontine cistern and cisterna magna were fenestrated, communicating the cyst with both cisterns. Ipsilateral cranial nerves 5-12 were visualized in the empty space without any compression. The rest of the cyst was delicately incised and removed in any area safely resectable, allowing for >80% cyst resection. Somatosensory evoked potentials/motor evoked potentials and the facial nerve were monitored intraoperatively without any permanent changes. After hemostasis and water-tight dural closure, the bone flap was resecured with a cranial plating system and the incision was sutured closed.

Postoperative course

**Figure 2:**
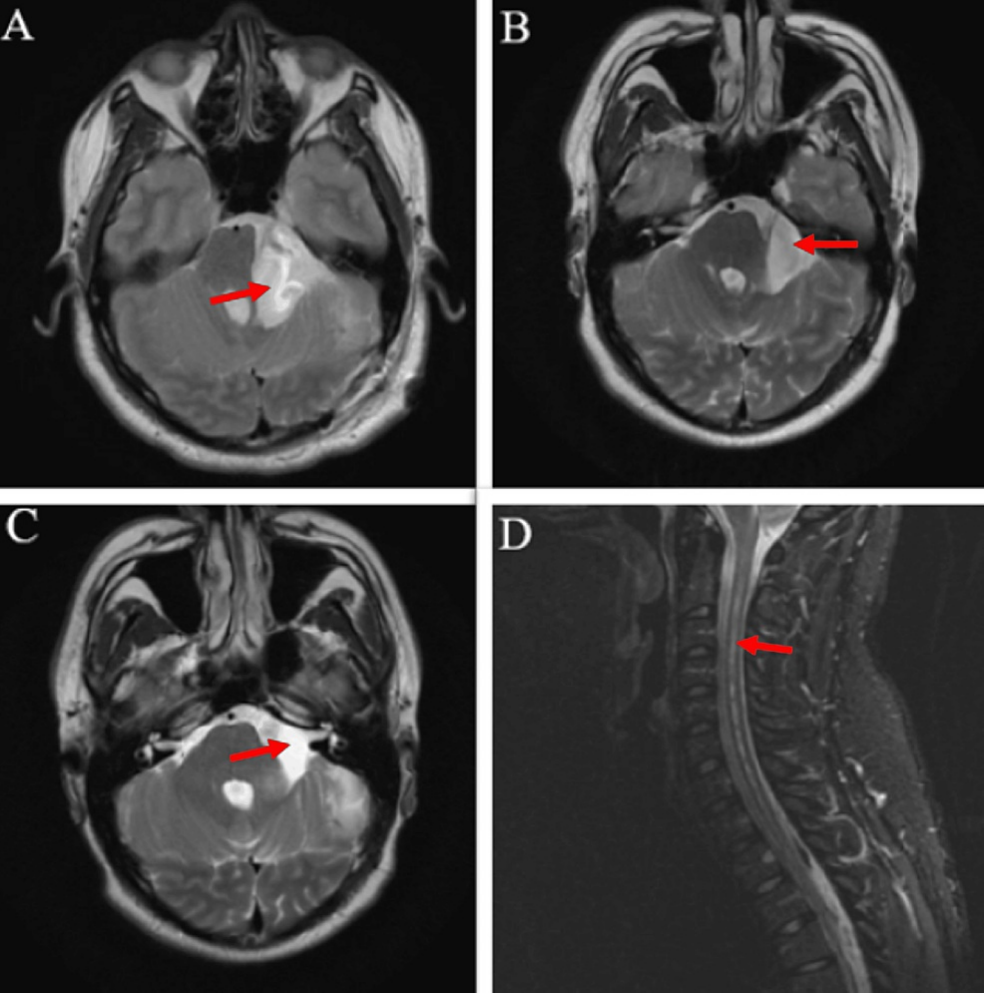
Postoperative MRI with arrows demonstrating improved PFAC, axial T2 MRIs (a) postoperative day 1; (b) six months postoperative; (c) 12 months postoperative; (d) sagittal T2 cervical MRI at 12 months postoperative, with arrow demonstrating the near-complete resolution of the syringomyelia

**Figure 3:**
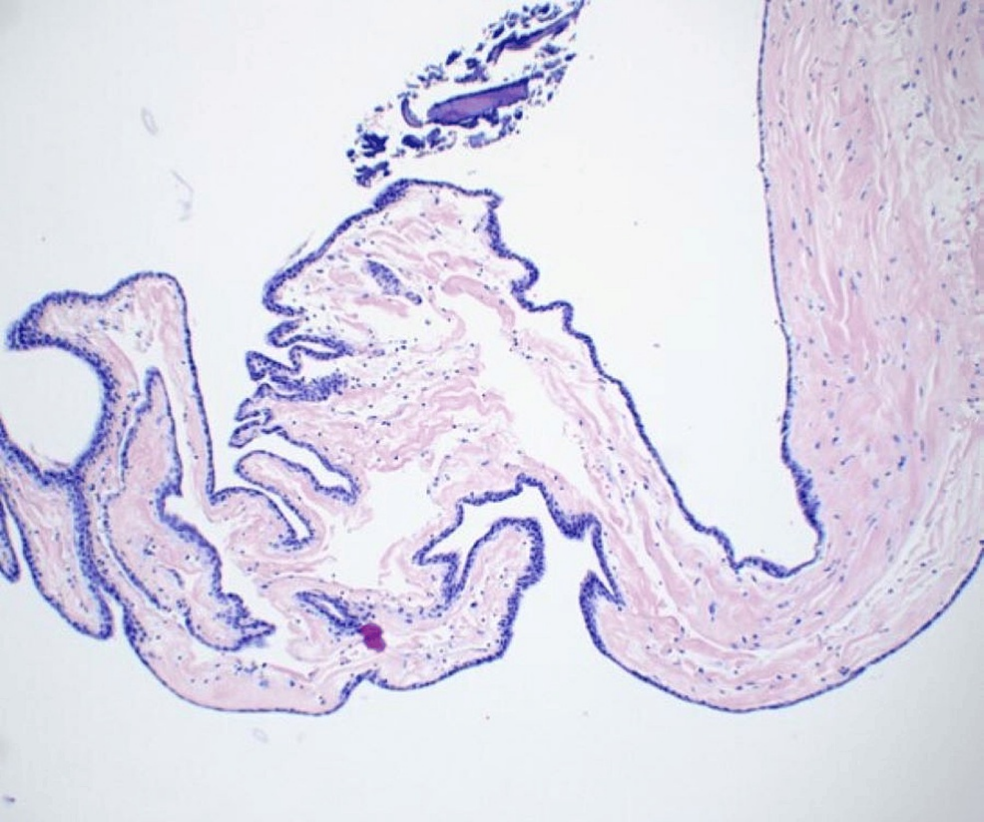
Hematoxylin and eosin stain demonstrating fragment consistent with benign arachnoid cyst. Keratin debris was also present (not within slide)

He had an uncomplicated postoperative course with immediate improvement in headaches. Immediate postoperative MRI demonstrated a decompressed posterior fossa with some persistent adherent cyst wall, edema in the brainstem, ventriculomegaly, and syringomyelia, all of which continued to improve over further imaging 12 months after surgery (Figure 2). Pathology was consistent with an arachnoid cyst, though scattered keratin debris was noted on pathology, representing a possible component of an indolent epidermoid tumor (Figure 3).

His follow-up three months later demonstrated complete resolution of his headaches without the need for any pain medication. He also had subjectively improved hearing by up to 60% compared to the unaffected ear. The patient denied any other cranial nerve deficits or cerebellar dysfunction. His subjective gait instability and recurrent falls progressively improved and were nearly resolved by a six-month postoperative follow-up. MRIs of the brain, cervical spine, and thoracic spine obtained six months after surgery demonstrated a persistent decrease in cyst size, resolution of tonsillar herniation, and near-complete resolution of the syringomyelia, which remained stable at 12 months postoperatively. At his most recent follow-up, 14 months postoperative, the patient had some minimal left lower extremity ataxia that was overall improved from his preoperative assessment.

## Discussion

This patient experienced remarkable improvement in hearing, which contrasts with most of the literature, in which patients with PFACs often do not experience much relief with their hearing loss [2-6]. In one case, a 29-year-old woman presented with 12 months of sensorineural hearing loss, which did not improve post-marsupialization on a three-month postop audiogram, despite good radiographic decompression of her PFAC and resolution of her ear pain [6]. In another case, a 52-year-old woman did not have significant improvement in her sensorineural hearing loss as measured by audiograms, despite cystoperitoneal shunt placement and radiographic decompression of her 3.0 x 1.7 cm CPA cyst [5]. A 67-year-old man experienced subjective improvement in hearing after a course of steroids, but left brainstem auditory evoked response (BAER) waves were absent. A PFAC was discovered and excised; however, this did not improve his BAER [3]. There are other similar cases [2,4]. On the contrary, one study did report five patients whose hearing loss improved (and one unchanged) [7], and another reported a 25-year-old woman whose sensorineural hearing loss completely resolved following resection [8]. It remains unclear why this study’s patient experienced relief, while many cases in the literature do not. The location and duration of symptoms seem to be unrelated based on this limited data. However, many of the studies fail to provide long-term outcomes or MRIs of the PFAC that may better delineate why hearing loss improves in certain situations. We hope this case will add to the literature by providing detailed pre- and postoperative MRIs, long-term outcomes, and unique histological findings. A limitation of this case is the lack of a formal audiogram, which would be helpful to better delineate the degree of hearing improvement. Many studies in the literature used audiograms to assess preoperative and postoperative hearing. Nonetheless, the subjective improvement of hearing combined with the improvements in headaches, gait disturbance, and radiographic findings supports the use of PFAC excision without the need for a ventriculoperitoneal shunt or other interventions.

The pathology, though consistent with a benign arachnoid cyst (immunohistochemistry was positive for pankeratin and EMA and negative for S100, synaptophysin, and PR), was also noted to have some keratin debris, typically consistent with epidermoid cysts. This was a unique finding given that preoperative imaging had no diffusion restriction that is commonly seen in epidermoid cysts. Epidermoid cysts are benign congenital tumors that develop from epithelial inclusions between the third and fifth weeks of gestation, when the neural tube is closing [9]. However, concomitant intracranial arachnoid and epidermoid cysts are not commonly reported in the literature. In a case by Kasliwal et al. [10], dual intracranial arachnoid and epidermoid cysts in the middle fossa are described. This may be underreported as many arachnoid cyst fenestration cases do not collect cyst walls for pathology, and this information is frequently excluded in the case series and reports published.

There is a scarcity of explicit symptomatic and radiographic outcomes in the literature; thus, it is difficult to assess if symptomatic improvement is related to a decrease in radiographic cyst size. Nonetheless, surgery may serve to reduce mass effect, intracranial pressure, and compression of the brainstem and cranial nerves. It should be noted that total resection is difficult as the cyst walls are often adherent to critical neurovascular structures; thus, combining fenestration seems to provide optimal outcomes.

Often, reports will describe that a patient “improved” but will not specify which symptoms improved. This is particularly a problem for patients who present with multiple symptoms or with studies that include multiple patients. Additionally, precise measurements of cyst sizes before and after surgery are inconsistently reported; this study aims to contribute to the field by setting a precedent for future reports. Nevertheless, PFACs are an esoteric pathology with a growing body of literature. Larger systematic reviews and meta-analyses will help clarify the role of surgery in this condition.

Limitations of this study include its inherent nature as a single case report, which restricts the generalizability of the findings to a broader population. The absence of comparative data, such as a control group, hinders the ability to determine the true effectiveness of the surgical intervention. While improvements in symptoms were objectively detected during physical examinations, the lack of standardized scales, such as audiograms for hearing assessment and reliable objective gait measures, limits the objectivity of the reported outcomes. Additionally, the relatively short follow-up duration of up to 14 months may not fully capture potential long-term outcomes and the recurrence of symptoms.

## Conclusions

PFACs are rare entities with a constellation of possible symptoms and may be asymptomatic. There is a deficit in long-term follow-up data on symptomatic and radiologic improvement. Surgical management remains contentious and is often up to the surgeon's discretion. This case report is novel in that it demonstrates that hearing may improve with PFAC fenestration and should be explored further.
